# Gratuitous Treatment at Clinics

**Published:** 1884-01

**Authors:** 


Gratuitous Treatment at Clinics.—Frequent complaints have been made lately by young physicians of the injustice of the system of free treatment of diseases at dispensaries, etc., or rather of the abuse of that system, for there is naught but praise for institutions formed for the object of releaving suffering among the *poor*, and we cannot do else than re-echo these complaints and join in the crusade against a state of affairs by which money is taken from the pockets of worthy workers and placed in the hands of the well-to-do sick, and the fame of a few college professors enhanced. And if we are led to express these sentiments in regard to the case in human medicine, with what more reason do we do so in the case of veterinary medicine, for therein charity is not taken into consideration and made to "cover a multitude of sins." An animal must have an owner and the owner of an animal can hardly be said to be poor.

The subscription system, by which sick animals can be treated on their owners paying a nominal sum annually, as adopted in some veterinary colleges, is but a step removed from the gratuitious one and as fully keeps from the general practitioner of veterinary medicine cases which otherwise would come to him in the course of his legitimate business. C.

